# Prevalence of Oropharyngeal Dysphagia in Adults With Type 2 Diabetes Mellitus

**DOI:** 10.7759/cureus.103712

**Published:** 2026-02-16

**Authors:** Astrid E Rosero-Castillo, José Rosmal Cortés Ponce, Marco Antonio Mendez Saenz

**Affiliations:** 1 Otolaryngology - Head and Neck Surgery, Universidad Autónoma de Nuevo León, La Facultad de Medicina y Hospital Universitario "Dr. José Eleuterio González", Monterrey, MEX

**Keywords:** diabetic neuropathy, eat-10, oropharyngeal dysphagia, screening tool, swallowing impairment, type 2 diabetes

## Abstract

Introduction

Type 2 diabetes mellitus (T2DM) is a metabolic disease with high prevalence and multiple complications, including diabetic neuropathy, which can interfere with the swallowing process, causing dysphagia and increased morbidity and mortality. The Eating Assessment Tool-10 (EAT-10) questionnaire is a validated and sensitive tool for the clinical screening of dysphagia in asymptomatic population.

Materials and methods

This is a descriptive and prospective study that included 69 patients over the age of 18 diagnosed with T2DM, with no neurological or surgical history affecting swallowing. The validated Spanish version of the EAT-10 questionnaire was administered, considering dysphagia to be a score ≥3. Demographic variables, duration of diabetes, and treatment were recorded and analyzed using inferential and descriptive statistics with a significance level of p≤0.05.

Results

A total of 69 patients were involved in the study, 44 (63.8%) of whom were women. The mean age was 54.7 years and the average duration of T2DM was seven years. A prevalence of dysphagia was found in 16 patients (23.2%), with no significant difference between this diagnosis and the variables of age, sex, or duration of the disease (p≤0.05). The most frequently affected items on the EAT-10 were those related to effort when swallowing and a sensation of residue in the throat.

Conclusion

Nearly a quarter of the patients with T2DM evaluated showed signs consistent with oropharyngeal dysphagia. The results reinforce the value of the EAT-10 as a rapid, inexpensive, and noninvasive screening tool for detecting subclinical swallowing disorders in the diabetic population.

## Introduction

Diabetes mellitus (DM) is a disease characterized by high blood glucose concentrations related to an imbalance between gluconeogenesis and glycogenolysis. In type 2 diabetes (T2DM), it is secondary to non-autoimmune β-cell dysfunction [1], with a prevalence of 11.1% and is identified by the World Health Organization (WHO) as the eighth leading cause of death globally [2,3].

The complications associated with this endocrinopathy have been categorized as microvascular, such as peripheral neuropathy, nephropathy, and retinopathy; and macrovascular, like cardiovascular disease, peripheral arterial disease, and stroke [4]. Diabetic neuropathy is believed to affect almost 50% of individuals with DM during their lifetime. It is caused by prolonged hyperglycemia, which results in oxidative stress to the peripheral nerves, damaging their ability to conduct motor and sensory impulses, as well as affecting the autonomic system, impairing sympathetic and parasympathetic conduction. The symptoms and signs will depend on the organ involved [5,6].

Swallowing is a vitally important process that allows food and liquids to be ingested efficiently and safely, enabling normal physiological functions to be maintained. It consists of four phases, the first two of which are voluntary and the last two involuntary. First preparatory oral phase, second propulsive oral phase, third pharyngeal phase, and fourth esophageal phase. The mechanisms that enable normal swallowing are complex and multifactorial, as the pharynx plays a dual role in both respiration an deglutition. Effective coordination of these two functions is crucial and depends on the synchronized sensorimotor activities mediated by cranial nerves V, VII, IX, and X [7-10].

Dysphagia is defined as a disruption of the normal swallowing process, is a frequent and serious complication of many neurological conditions. It varies when one or more elements of the swallowing system are impaired and represents a complex syndrome that WHO describes as the inability to transfer the bolus safely and efficiently from the oral cavity to the esophagus. Patients diagnosed with dysphagia may experience dehydration, malnutrition, and an increased risk of pneumonia, which is associated with higher rates of hospital admission, the need for long-term care, and mortality [9-15].

The prevalence of dysphagia associated with this endocrinopathy is estimated to be between 5.4% and 27%, closely related to the presence of diabetic neuropathy, which is among the most frequent complications associated with diabetes mellitus [16-21]. There are various ways to detect and evaluate symptoms related to dysphagia, including targeted tests such as fiberoptic endoscopic evaluation of swallowing (FEES) or videofluoroscopy (VFSS), which are invasive. However, there are also tools such as standardized questionnaires that are easy to use and non-invasive [22-24].

Among the various tools available for dysphagia screening, the Eating Assessment Tool-10 (EAT-10), validated by Belasfky et al. in 2008, is a self-administered instrument composed of 10 items with a score ≥3 as an indicative threshold for dysphagia. It is non-invasive, easy to administer, and validated in multiple clinical contexts. The correlation between the results of the EAT-10 and instrumental swallowing tests such as the VFSS and FEES shows good agreement with the objective findings of penetration, aspiration, and pharyngeal residue [25-27].

## Materials and methods

Study design: descriptive and prospective

Participants and Setting

A total of 69 adult patients between the ages of 18 and 65 with a known diagnosis of T2DM and treated at a tertiary hospital in Monterrey, Mexico, between October 2024 and October 2025, were included in the study. The following were excluded: people over the age of 65, people under the age of 18, patients with comorbidities or surgical history affecting swallowing, such as stroke, progressive neurological disease, head and neck cancer, and patients without type 2 diabetes mellitus.

This study adhered to the principles of the Declaration of Helsinki, the Belmont Report, the Nuremberg Code, and the provisions of Mexican Official Standard NOM-012-SSA3-2012. Ethical approval was obtained from the Ethics Committee of Dr. Jose Eleuterio González Hospital under registration code OT24-00009 in May 2024.

Data Collection

A brief interview was conducted to obtain demographic and disease data, including age, sex, duration of T2DM, and type of therapy (insulin or oral medication). Information was also collected on medical history to rule out any systemic disorders related to oropharyngeal dysphagia.

The EAT-10 [25] questionnaire was administered, which uses 10 items to assess the patient's perception of the severity of symptoms related to dysphagia. Each item is rated on a Likert scale from zero to four points, where zero indicates no symptoms and four represents maximum severity. The total score is obtained by adding the values of the 10 items, with a score ≥3 indicating dysphagia. 

Statistical Analysis

Frequencies and percentages were obtained for categorical variables, while numerical variables were described using measures of central tendency and dispersion (median, interquartile range or IQR). The Shapiro-Wilk test was used to evaluate data normality. Comparisons of numerical variables between independent groups were performed using the Mann-Whitney U test. Spearman's correlation coefficients were calculated to determine the degree of association between numerical variables. A p-value ≤0.05 was considered statistically significant. All analysis was conducted using the IBM SPSS Statistics for Windows, Version 25 (Released 2017; IBM Corp., Armonk, New York, United States).

## Results

We analyzed a total of 69 patients, 44 (63.8%) of whom were women. The median age was 56 years (IQR 48-61.5). The median duration of diabetes was seven years (IQR 4-14). Most patients (n=66; 95.7%) were receiving pharmacological treatment for diabetes, and only two patients (2.9%) had micro- or macrovascular lesions. The median score on the EAT-10 scale was zero (IQR 0-3), as shown in Table 1.

**Table 1:** Demographic and clinical characteristics of the patients IQR: Interquartile range.

	Total patients (n=69)	Without dysphagia (n=53)	With dysphagia (n=16)	p-value
Women n (%)	44 (63.8)	32 (60.4)	12 (75)	0.286
Age median (IQR)	56 (48-61.5)	56 (49-62)	56.5 (45.25-60.5)	0.355
Diabetes time evolution median (IQR)	7 (4-14)	8 (4.5-14)	5 (1.25-15.25)	0.318
Pharmaceutical treatment, n (%)	66 (95.7)	50 (94.3)	16 (100)	0.999
Micro/macrovascular damage, n (%)	2 (2.9)	2 (3.8)	0	0.999

The prevalence of dysphagia was 23.2% (95% CI: 14.5-34.1%). No significant correlations were observed between the duration of diabetes and the EAT-10 score, nor between age and the EAT-10 score. Likewise, no significant associations was observed between age and the presence of dysphagia or between diabetes duration and dysphagia, as shown in Table 2.

**Table 2:** Pevalence of dysphagia and its assosiation with clinical variables All correlations were nonsignificant (p≥0.05); EAT-10: Eating Assessment Tool-10.

Outcome	Results
Prevalence of dysphagia	23.2% (95% CI: 14.5-34.1)
Correlation between diabetes duration and EAT-10 score	r= -0.081, p=0.509
Correlation between age and EAT-10 score	r= -0.191, p=0.136
Association between age and dysphagia	p=0.355
Association between diabetes duration and dysphagia	p=0.318

**Figure 1:**
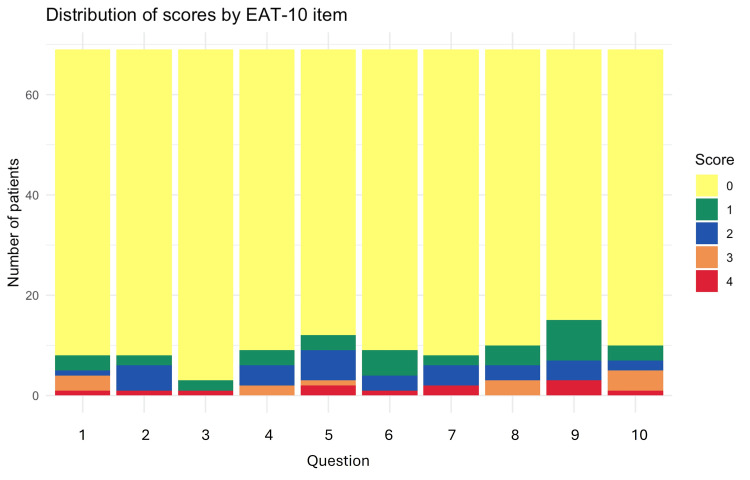
EAT-10 score distribution EAT-10: Eating Assessment Tool-10

Analysis of the EAT-10 scale items showed that most patients had low scores, reflecting a median total score of zero. However, some items showed greater impact, such as the item “swallowing is stressful,” which was the most compromised, with five patients achieving scores ≥3, followed by the items “swallowing pills takes extra effort,” “when I swallow food sticks in my throat,” and “I cough when I eat,” with three patients each with high scores. The distribution by scores is shown in Figure 1.

## Discussion

This study evaluated the prevalence of dysphagia in patients with T2DM using the EAT-10 scale. The findings suggest that, although most patients do not present obvious symptoms, there is a subgroup that shows impairment in specific aspects of swallowing, which may go unnoticed if a systematic evaluation is not performed.

The prevalence of dysphagia found in this sample is in line with previous studies, which estimate that up to a quarter of patients with T2DM may have dysphagia [16,28]. This indicates that, although not all patients develop clinically obvious symptoms, dysphagia is a relatively common complication that warrants attention.

A study conducted by Zakaria et al. [16] evaluated swallowing function in patients with diabetes who reported self-declared discomfort, using FEES. The authors observed that patients with high scores on the EAT-10 scale, despite having a swallow considered “safe” in terms of aspiration according to the penetration-aspiration scale, showed alterations in laryngeal sensation, presence of residual secretions, and bolus retention in the pharynx.

These findings suggest a decrease in swallowing efficiency, even in the absence of obvious aspiration. These data are particularly relevant, as they indicate that dysphagia in T2DM does not always manifest as aspiration, but can present as a subclinical loss of swallowing coordination and efficiency, which is easily overlooked if the assessment is limited to patient-reported symptoms.

No significant correlations were observed between age, duration of diabetes, and EAT-10 scores, which is consistent with previous reports in which the onset of dysphagia was not directly related to these demographic factors [29]. However, the literature suggests that diabetic neuropathy, especially autonomic neuropathy, can affect oropharyngeal and esophageal function, contributing to the onset of dysphagia even in the early stages of the disease [30].

The finding of a subgroup with high scores on specific items of the EAT-10 emphasizes the importance of early detection. Dysphagia can have significant consequences, including risk of aspiration, malnutrition, and impaired quality of life [14,15]. For this, reason, it is important to implement routine screening strategies in patients with T2DM, even in those without obvious symptoms. Validated tools such as the EAT-10 can help identify patients at risk in a simple and efficient manner, facilitating early care from swallowing specialists.

Early detection of dysphagia not only allows for timely intervention to prevent complications but can also serve as an indicator of subclinical neurological involvement in patients with diabetes. Incorporating routine swallowing assessments into primary care and ongoing management of T2DM patients may enhance quality of life and help reduce the morbidity and mortality associated with this condition.

An example of the clinical value of systematic dysphagia screening is provided by the study by Aghili et al., who used both self-administered questionnaires and objective functional tests, including the Mann Assessment of Swallowing Ability (MASA) [28]. The authors showed that patients with diabetes experience a significant deterioration in quality of life related to swallowing disorders, with a higher prevalence among women and those undergoing insulin treatment. These findings reinforce the need to routinely incorporate dysphagia screening instruments into the follow-up of patients with diabetes, even in the absence of overt symptoms. This is in order to promote early detection and prevent nutritional and respiratory complications associated with this dysfunction.

Among the limitations of the study are the small sample size and the geographical concentration of participants, which could limit the generalizability of the findings. In addition, the assessment of dysphagia was based exclusively on the EAT-10 scale, without the application of objective tests, which could underestimate or overestimate the actual prevalence of the condition.

## Conclusions

The findings suggest that dysphagia is a common but often underdiagnosed complication in patients with T2DM. The implementation of systematic screening and early detection using validated tools is essential to identify patients at risk, prevent complications, and optimize comprehensive clinical management. The use of the EAT-10 is simple and easy during consultation, and for diagnosis and follow-up. Integrating periodic swallowing assessments into primary care for these patients could improve quality of life and reduce the morbidity and mortality associated with this condition.
